# A novel environmental nano-catalyst of zeolite amended with carbon nanotube/silver nanoparticles decorated carbon paste electrode for electro-oxidation of propylene glycol

**DOI:** 10.1038/s41598-022-12268-4

**Published:** 2022-06-01

**Authors:** Soha A. Abdel-Gawad, Amany M. Fekry

**Affiliations:** Chemistry Department, Faculty of Science, Cairo University, Giza, 12613 Egypt

**Keywords:** Chemistry, Catalysis, Energy

## Abstract

A novel environmental nano-catalyst based on zeolite (ZE) adjusted with carbon nanotube/silver nanoparticles (Ag/CNT) ornamented carbon paste electrode (CPE) is used for electrochemical oxidation of propylene glycol (PG) in 0.5 M H_2_SO_4_ solution. The techniques like cyclic voltammetry (CV), chronoamperometry (CA) and electrochemical impedance spectroscopy (EIS) are utilized to achieve the catalytic activity performance. Surface characteristics are achieved by means of scanning electron microscope (SEM) and Energy dispersive X-ray analysis (EDX) techniques. Enhancing the loading magnitude of CNT into catalyst's ingredient can meaningfully develop the catalytic activity of the electrocatalyst towards propylene oxidation. The impact of altering the concentration of propylene glycol and the scanning rate on the resulting electrocatalyst performance during the oxidation cycle is considered. Chronoamperograms present an amplify of the steady state oxidation current density values after addition of these nano-catalysts. A promising catalytic stability of nano-catalyst has been achieved in electing its use for propylene glycol electro-oxidation in fuel cells applications.

## Introduction

The world has faced energy shock because fossil fuel reserves are rapidly exhausted. Also, the utilization of fossil fuels is being perceived for its harmful influences that may cause a natural and social crisis^1,2^. Thus, it is important to look for a practical alternate fuel and make new energy conversion instruments to resolve these energy cohort difficulties^3,4^. Direct alcohol fuel cells dependent on small organic molecules have great consideration as probable power devices, mainly for portable electronic devices and electric vehicles in view of their high energy density, efficiency, low ecological contamination and low working temperature^5,6^. The electrocatalytic oxidations of different small organic molecules on various edited electrodes have been examined for application in fuel cells^7–13^. Most of recent reports, polyalcohol such as ethylene glycol, propylene glycol and glycerol, are a good choice in fuel cells due to its high reactivity in electro-oxidation, inflammability, lower toxicity and high boiling point that allows tranquil handling and storage^14,15^. Polyalcohol fuels are valuable due to they can be produced from renewable sources^16,17^. Furthermore, to improve the efficacy of fuel employment, active catalysts, having enough ability to break strong C–C bond in polyalcohol are highly wanted^18–20^.The electrochemical oxidation of polyalcohol on noble metals such as platinum, palladium, and gold has been demonstrated in several recent investigations. Sadiki et al.^21^ have studied the polyalcohol oxidation in alkaline media by improving the efficacy of the palladium catalysts edited by several adatoms (antimony, tin, lead). The existence of bimetallic surface leads to enhance the oxidation rate of these alcohols. Munoz et al.^22^ presented that a carbon-supported Pd-Cu nanocatalyst can increase the polyalcohol oxidation rate in alkaline medium. The developments have been accredited to both electronic and bifunctional effects. Furthermore; the Cu/Pd ratio seems to have a noteworthy impression on the rate of oxidation^23,24^. To make the electro-oxidation of polyalcohol practically valuable, novel inexpensive and selective catalysts are required. Catalyst plays a crucial role in electrochemical energy conversion in fuel cell. Carbon paste electrode (CPE) is the most popular catalytic support material due to their strong electronic conductivity, comparative stability in different media and high effective surface area^25–27^.The nano-modified electrodes are substantial to improve the catalytic activity in order to reduce production costs.

Recently, silver nano particles (AgNPs) have potentially demonstrated in electrocatalysis applications. AgNPs can act as a conduction center to improve the transmission of electrons and deliver exceptional catalytic activity^28,29^. Carbon nanotubes (CNTs) are porous constituents with special characteristics, high efficiency, high surface area, strong conductivity, and chemical stability^30^. Carbon nanotube has high electrocatalytic activity in numerous substances and therefore suitable for building various electrocatalyst with high efficiency^31–34^. Zeolites are known as microporous crystalline aluminosilicate materials with high surface area, adsorptive and molecular sieving materials. Zeolites are unique materials, which can be exploited in the development of modified electrodes with high chemical and thermal stability^35–38^. Nevertheless, the propylene glycol oxidation in acidic media is quiet not fully revealed and requires to be studied. It is well recognized that carbon paste electrode with nano-material showed electrocatalytic act towards propylene glycol oxidation in acidic medium due to strong adsorbability of PG molecules on the nano-material surface.

The aim of this work is to produce a novel environmental nano-catalyst created from modified carbon paste electrode with zeolite, carbon nanotube and silver nanoparticles to have the collective characteristics of the three modifiers together. After analysis of the electrochemical response of this novel catalyst using various techniques, it was found to give high electrocatalytic activity towards electrooxidation of propylene glygol. A different percentages of the catalyst with large surface area, simple and low cost construction are recommended for propylene glycol oxidation in acidic medium.

## Experimental

### Materials and reagents

The chemicals are graphite, multi walled carbon nanotube, zeolite Y-type (SiO_2_/Al_2_O_3_- ≥ 5.1) and silver nanoparticles (AgNPs) powder (dispersion nanoparticles, < 100 nm), propylene glycol (99. 8%), Sigma–Aldrich (USA) and were used without further purifications. All solutions are prepared utilizing triple distilled water. All experiments were achieved at room temperature. The solution pH for propylene glycol is 3.9 before adding the acid. After adding the sulphuric acid, the pH value for different concentrations of acid is in the range of 0.1–0.4.

### SMWZCPE modified electrode preparation

In order to develop the catalytic activity of (CPE) electrode different weights of zeolite and silver nanoparticles were investigated with no significant change in current. However, the loading of CNT into the catalyst (10–40 mg) gives a significant change in current and was considered in Table 1.

**Table 1:** Experimental condition for the synthesis of catalysts.

Types of catalysts	Zeolite (mg)	AgNPs (mg)	CNT (mg)
Electrode 1 (Ecat-1)	100	5	40
Electrode 2 (Ecat-2)	100	5	30
Electrode 3 (Ecat-3)	100	5	20
Electrode 4 (Ecat-4)	100	5	10

The best modification for carbon paste (CPE) electrode was made by mixing 5 g of graphite powder with drops of paraffin oil on a glassy mortar, then 40 mg of carbon nanotube, 5 mg of silver nanoparticles and 0.1 g of zeolite were added to set up the catalyst (Ecat-1) (SMWZCPE), which was utilized in all experiments. The carbon paste was packed in a Teflon tube with pressing to acquire a smooth surface.

### Cell and apparatus

A three-electrode cell enclosing a platinum rode as a counter electrode (CE), saturated calomel electrode (SCE) as a reference electrode (RE) and (SMWZCPE) as the working electrode (WE) was used. Cyclic voltammetry (CV), Chronoamperometry (CA) and Electrochemical impedance spectroscopic (EIS) measurements are achieved by EC-Lab SP 150 Potentiostat electrochemical workstation. EIS are done at 10 mV ac amplitude at frequency of 1.0 mHz to 100 kHz. EC-Lab software is operated for demonstrating. The electrochemical measurements were performed at room temperature. Scanning electron microscopic (SEM) measurements were performed by SEM Model Quanta 250 FEG (Field Emission Gun) related with EDX Unit (Energy Dispersive X-ray Analyses) (FEI company, Netherlands). To determine the sample's specific surface area and pore-size distributions, the Brunauer–Emmett–Teller (BET) and Barrett-Joyner-Halenda (BJH) procedures were utilised.

## Results and discussion

### Characterization of SMWZCPE modified electrode

**Figure 1:**
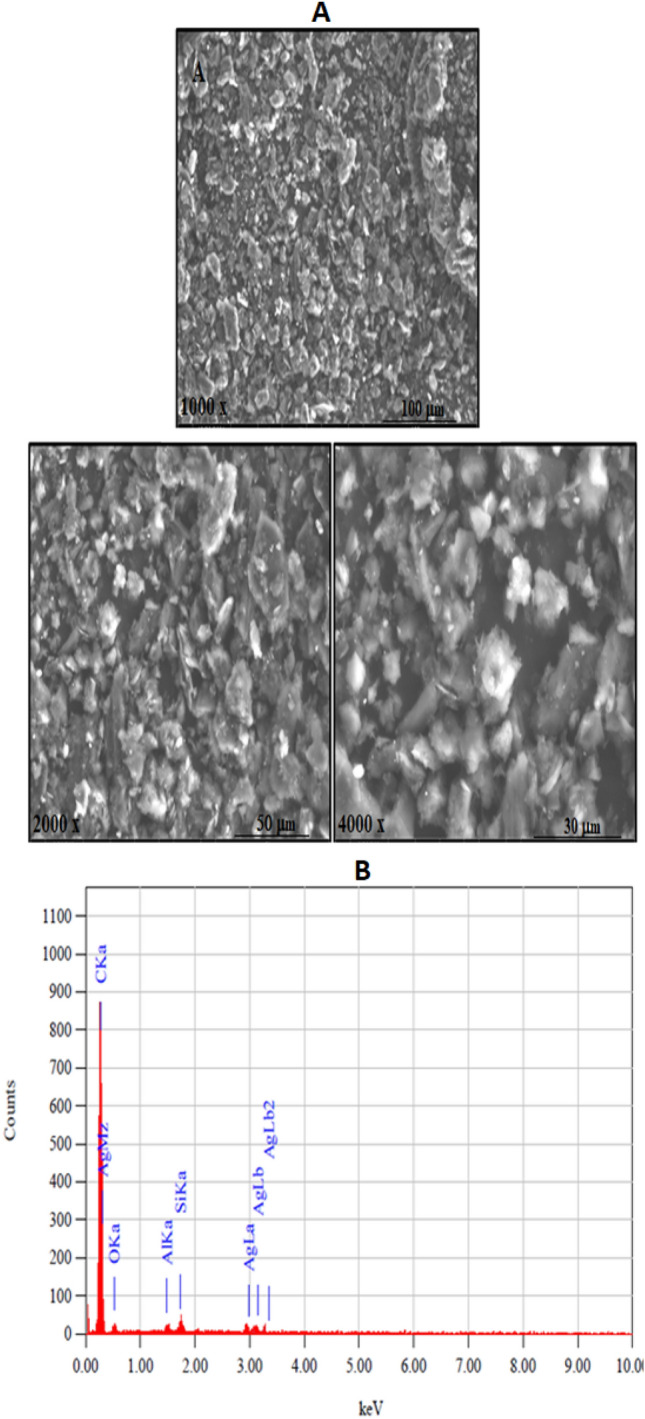
(**A**) SEM images for SMWZCPE modified electrode with different magnifications. (**B**) EDX analysis for SMWZCPE modified electrode.

Figure 1A shows the SEM images of the SMWZCPE modified electrode which reveals that silver nanoparticles, CNT and zeolite are dispersed well in a uniform distribution with the graphite paste. The rough surface with vertically arranged nanoarrays can be detected in Fig. 1A. Thus, the structural advantages of nanomaterial as being near ideal candidates to be significant catalyst with low electron affinity, high electron mobility, and outstanding chemical and physical stability and highly conductive. It can be applicable in nanoscale molecular electronics, sensing and actuating devices, or as reinforcing additive fibers in functional composite materials^39^. Generally, constructing nanostructured cartalysts play a substantial role in exploiting promising supercapacitors with satisfactory electrochemical performance^40^. Figure 1B demonstrates well the EDX spectrum of SMWZCPE which approves the existence of C, O, Ag, Al and Si by a good percentage in the formed electrocatalyst. The uniform spreading of the elements along the entire catalyst surface offered an opportunity to improve the active electrochemical surface area and increasing the elecrocatalytic efficiency.

**Figure 2:**
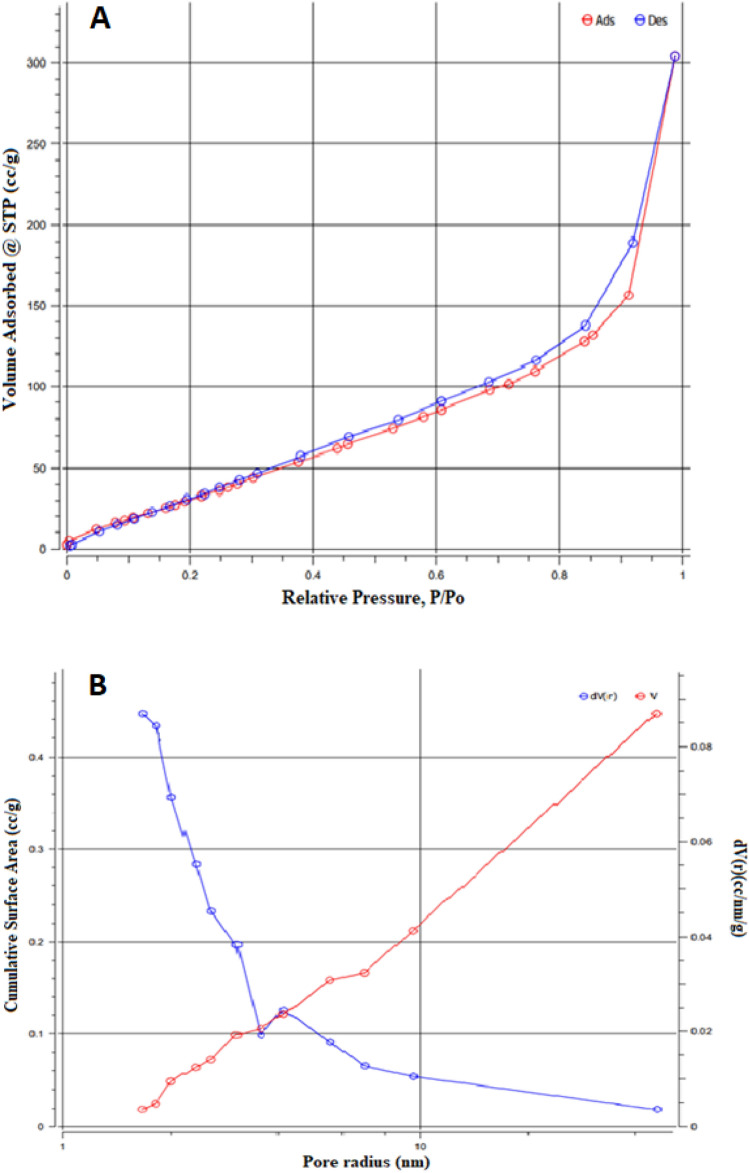
(**A**) N_2_ adsorption–desorption isotherms, (**B**) pore size distributions using quenched solid density functional theory (QSDFT).

The adsorption–desorption isotherms and pore size distribution of the nano-catalyst based on zeolite (ZE) adjusted with Ag/CNT ornamented carbon paste electrode are displayed in Fig. 2A and B, respectively.

Under relatively low pressure (P/Po < 0.01), the adsorbed amount of N_2_ presented a vertical rise for all materials, signifying the existence of a signicant amount of micropores. Also, it is clear from increasing the mesoporous structure that there is accessible pathways for the diffusion and absorption of ions. Generally, the measured specific surface area using BET is found to be 139 m^2^ g^-1^ with an average pore size of 7 nm indicating the existence of a signicant amount of micropores. The higher specifc surface area measured in is attributed to the proper proportion of the micropore volume and mesopore volume^41^.

### Electrocatalytic properties of SMWZCPE modified electrode

**Figure 3:**
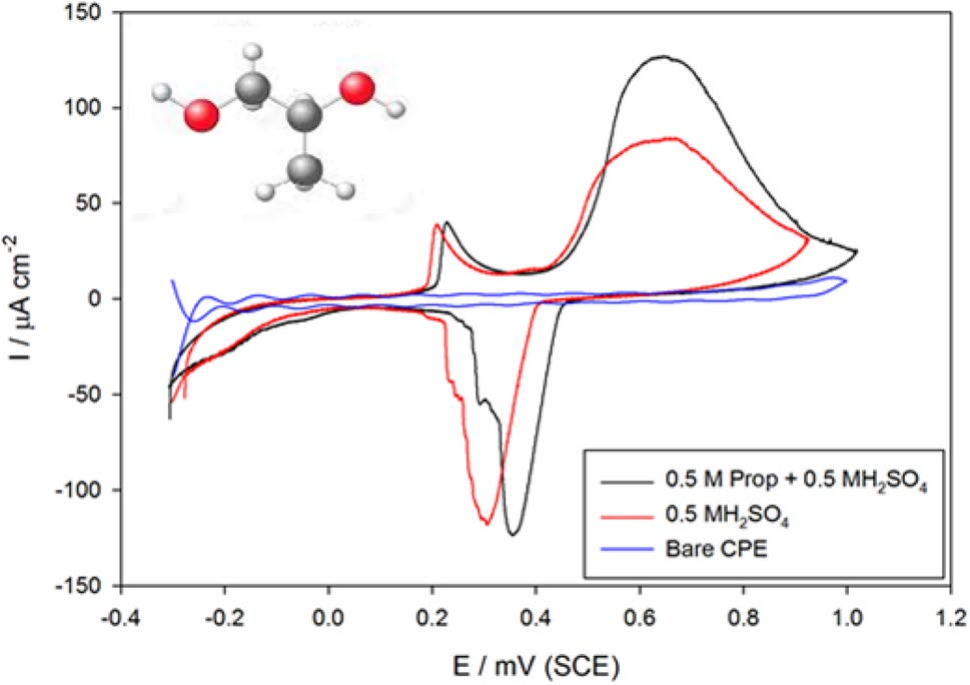
CVs of bare CPE in 0.5 M H_2_SO_4_ and SMWZCPE in 0.5 M H_2_SO_4_ with and without 0.5 M propylene glycol at scan rate of 100 mV·s^-1^.

Figure 3 presents the CVs obtained at the bare carbon paste electrode in 0.5 M H_2_SO_4_ of pH 0.3 and modified electrode SMWZCPE in 0.5 M H_2_SO_4_ with and without 0.5 M propylene glycol at a scan rate of 100 mV·s^-1^. CV verified at the bare electrode seemed unremarkable indicated catalytic inactivity. However, the CVs of the modified electrode offered a developed electro-catalytic activity towards propylene oxidation. The oxidation of propylene at modified electrode reveals a reversible oxidation peak at 0.7 V and 0.38 V with a peak current ~ 50 times more than the bare one. This indicated that the electrocatalytic activity of the modified electrode surface can be improved upon addition of zeolite, silver nanoparticles to the carbon nanotube matrix where a large effective surface area was obtained inducing higher adsorptivity of propylene through active hydroxyl function group to the modified electrode surface.

**Scheme 1:**
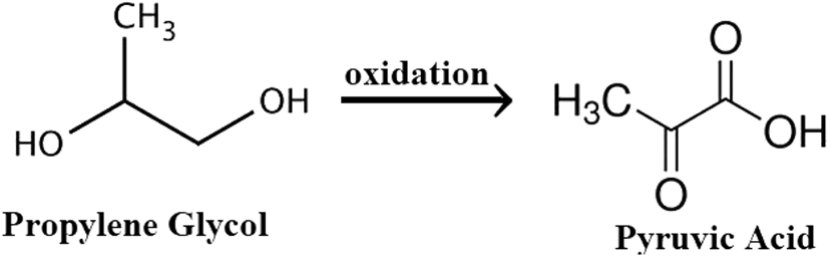
Propylene glycol oxidation.

The propylene glycol oxidation is suggested in Scheme 1 to be as follows:

As shown in Scheme 1, propylene glycol is simply oxidized to pyruvic acid (2-oxopropanoic acid) containing carbonyl and carboxylic groups. This acid is highly applicable for producing fine chemicals and pharmaceuticals, which can be acquired by propylene glycol oxidation^42^.

**Figure 4:**
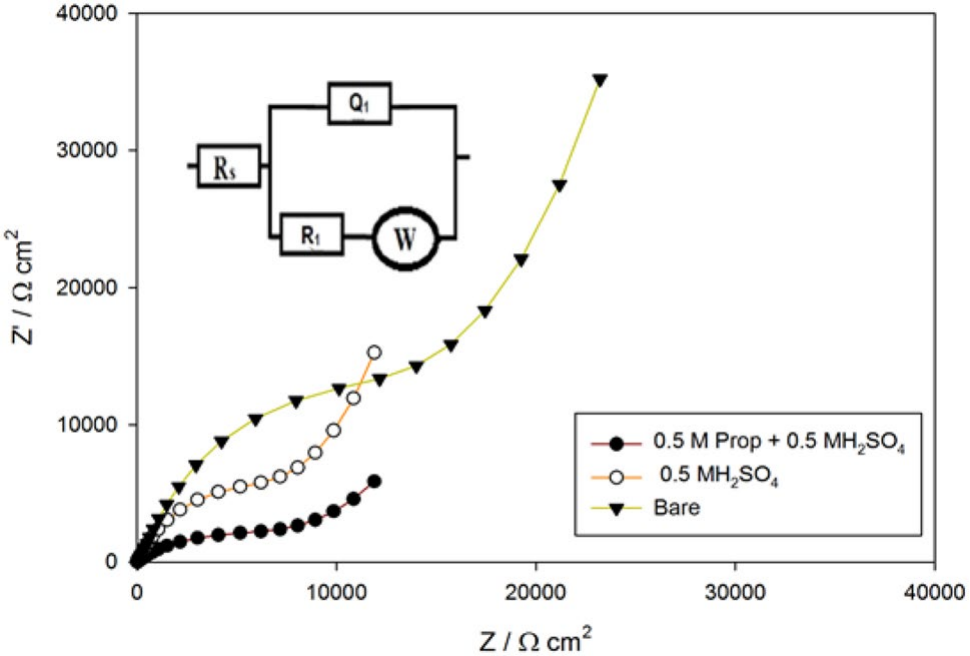
Nyquist plots of bare CPE in 0.5 M H_2_SO_4_ and SMWZCPE in 0.5 M H_2_SO_4_ with and without 0.5 M propylene glycol. (Inset: Fitting model (simple Randles model)).

Electrochemical impedance spectroscopy technique is applied to examine the conductivity (catalytic activity) of the modified electrode towards propylene oxidation which is inversely proportional to the impedance. EIS scans distinguished at the peak potential 0.7 V for bare in 0.5 M H_2_SO_4_ and SMWZCPE electrode in 0.5 M H_2_SO_4_ with and without 0.5 M propylene glycol. Figure 4 as Nyquist plots showed a semi-circle links to a charge transfer resistance and a line links to a diffusion process at both high and low frequencies, respectively. The experimental results were fitted with one-time constant model (Fig. 4 inset) including R_s_ (solution resistance), R_1_ (charge transfer resistance), W (Warburg impedance linked to diffuesion prcess) and Q_1_ (constant phase element of capacitance). Constant phase element was attributed to microscopic roughness and surface heterogeneity^43,44^. Bare electrode show a large semicircle diameter than that of modified SMWZCPE electrode signifing that impedance reduced and conductivity upsurges. These results support well the high oxidation peak current acquired from CVs response for modified SMWZCPE electrode.

The impedance (Z_CPE_) of a constant phase element is:

$${\text{Z}}_{{{\text{CPE}}}} = \left[ {{\text{C }}\left( {{\text{j}}\omega } \right)^{\alpha } } \right]^{{ - {1}}}$$

where α is an exponent account for surface heterogeneity, 0 ≤ α ≤ 1, j is the imaginary number (j = (− 1)^1/2^), ω = 2πf is the angular frequency in rad/s, f is the frequency in Hz = S^-1^^45-47^^.^ The EIS outcomes have confirmed the conclusions drawn from the above cyclic voltammetry experiments. The outcomes of EIS analysis are listed in Table 2. The outecomes certified well CV data, where SMWZCPE electrode have the highest current and lowest impedance values.

**Table 2:** Electrochemical impedance parameters**.**

Sample	R_1/_kΩ (cm^2^)	Q_1_ (μF)	α	W/k Ω (cm^2^ s^-1/2^)	R_s_/Ω (cm^2^)
Bare	19.2	11.1	0.78	9.8	21
SMWZCPE/0.5 M H_2_SO_4_	10.5	14.3	0.81	7.3	37
SMWZCPE/0.5 M prop + 0.5 M H_2_SO_4_	7.60	17.1	0.84	2.1	35

#### Effect of carbon nanotube content

**Figure 5:**
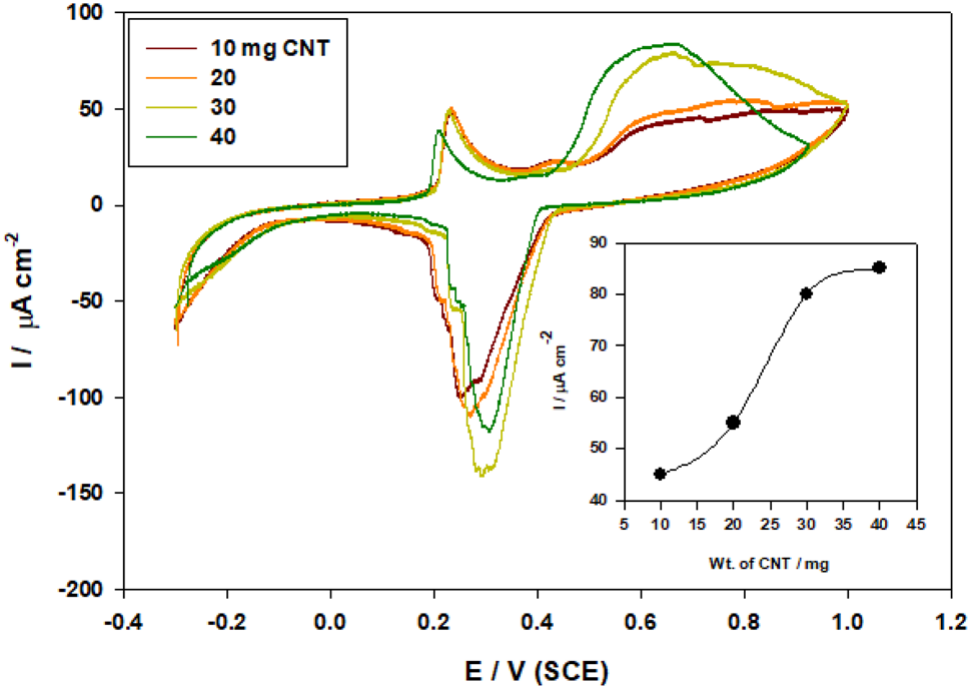
CVs of modified electrode SMWZCPE with various loading of CNT (10–40 mg) in 0.5 M H_2_SO_4_ at scan rate of 100 mV·s^-1^.

The loading of CNT into the catalyst ingredients has a substantial influence on the catalytic activity of the modified electerode towards propylene oxidation due to growth of surface area. Figure 5 displays the CVs respone for propylene oxidation on modified electrode SMWZCPE with various loading of CNT (10–40 mg) in 0.5 M H_2_SO_4_ at scan rate of 100 mV·s^-1^. The propylene oxidation is reliant on the loading amount of CNT and anodic peak current increase with the increase in CNT loading in the synthesized electrocatalyst (direct relationship). Also, the onset potential shifts to more negative values for the best performing. As clearly interpreted, the increase in anodic peak currents indicates a corresponding increase in avaliable active sites with higher adsorption extent for hydroxyl group, which required for propylene oxidation^31–33^.

#### Effect of scan rate

**Figure 6:**
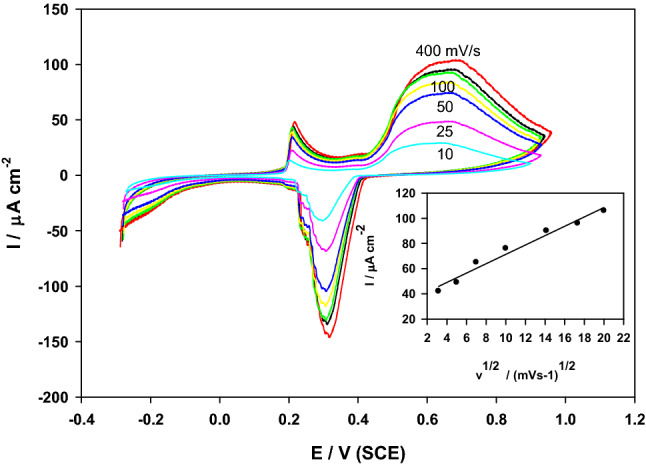
Effect of varying the scan rate from (10–400 mVs^-1^) on the anodic peak current of SMWZCPE in 0.5 M H_2_SO_4_. Inset: the plot of the anodic peak current and the square root of scan rate.

Effect of varying the potential scan rate (ν ranging from 10 to 400 mV·s^-1^) was performed on modified SMWZCPE electrode in 0.5 M H_2_SO_4_ in absence of propylene glycol to confirm the electrochemical activity of the catalyst in aqueous solution (Fig. 6). Increasing the scan rate resulted in higher anodic peak current density and positive shift occurs in the forward peak potential. A linear relationship between the anodic peak current and square root of the scan rate was gotten as shown in the inset of the figure, with the following equation:

$${\text{I}}_{{\text{p}}} \left( {\mu {\text{A}}} \right) \, = {34}.{13 } + { 3}.{71 }\nu^{{{1}/{2}}} \left( {{\text{mV}} \cdot {\text{s}}^{{ - {1}}} } \right)\left( {{\text{r}}^{{2}} = 0.{9766}} \right).$$

**Figure 7:**
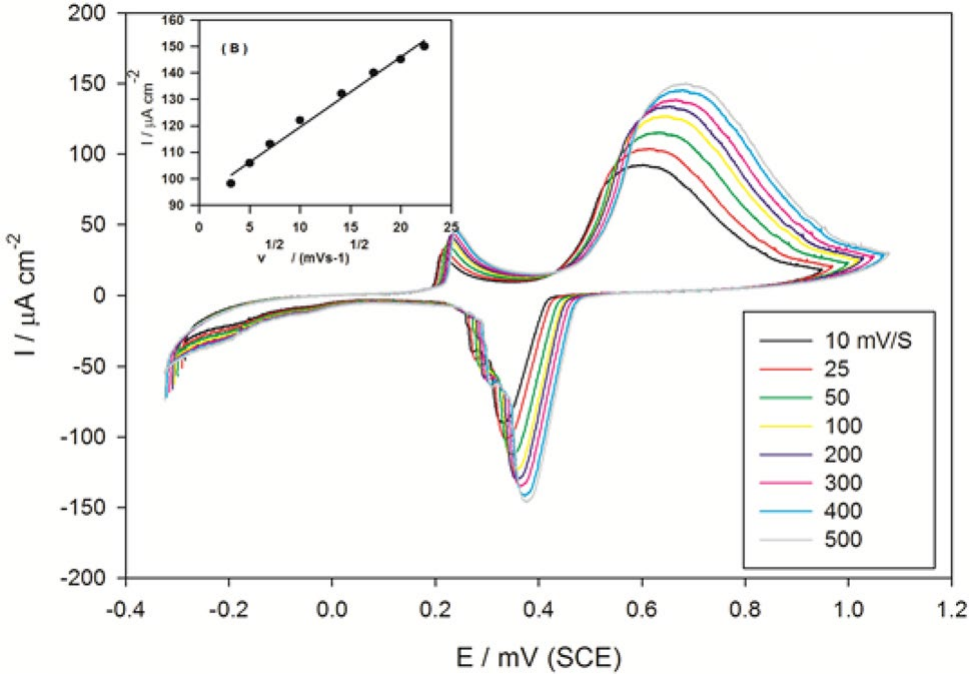
Effect of varying the scan rate from (10–500) mV·s^-1^ on the anodic peak current of SMWZCPE in 0.5 M H_2_SO_4_ with 0.5 M propylene glycol. Inset: the plot of the anodic peak current and the square root of scan rate.

The impact of the potential scan rate (ν ranging from 10 to 500 mV s^−1^) on the electrocatalytic anodic peak current of modified electrode SMWZCPE was also achieved in 0.5 M H_2_SO_4_ with 0.5 M propylene glycol and established in (Fig. 7). As the scan rate increases (10-500 mV s^−1^), the oxidation peak current amplified constantly and the peak potential moved positively. The plot of anodic peak current and square root of the scan rate (Inset B) leads to a linear relation: I_p_ (μA) = 91.22 + 2.77 ν ^1/2^ (mV s^−1^) (r^2^ = 0.9466), which approves that the oxidation process of propylene glycol is diffusion controlled mechanism with some adsorption phenomena^48,49^.

#### Effect of propylene glycol concentration

**Figure 8:**
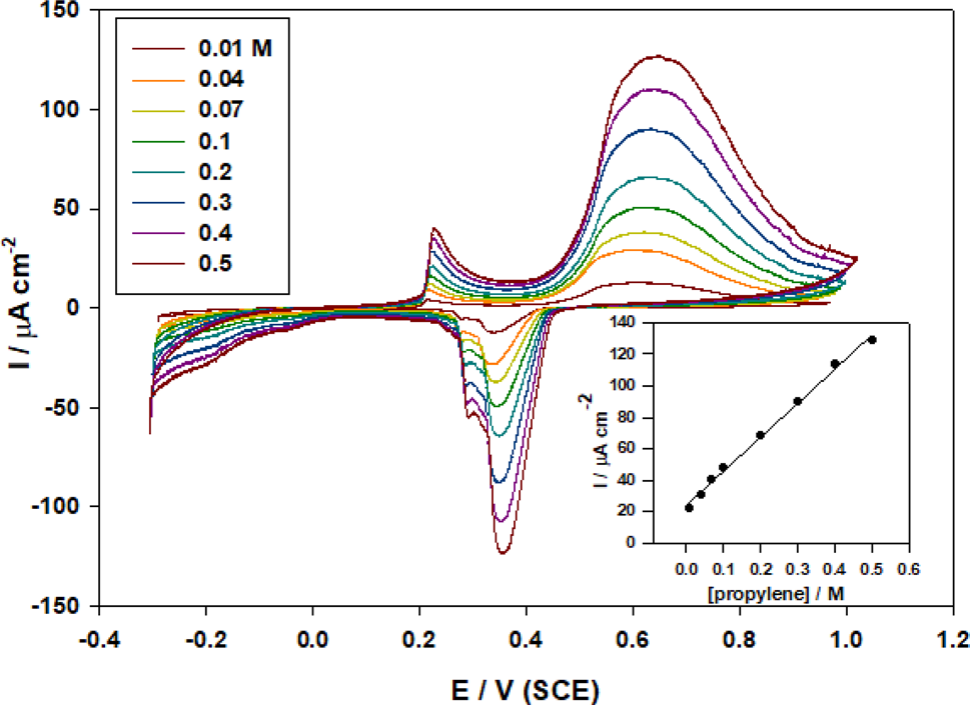
Effect of varying the propylene glycol concentration from (0.01- 0.5M) on the anodic peak current of SMWZCPE in 0.5 M H_2_SO_4_. Inset: the plot of the anodic peak current and the propylene glycol concentration.

The synthesized electrode was applied to distinguish the impact of propylene concentration for fuel cells application. The oxidation of propylene in the range of 0.01 to 0.5 mol/L was considered on this electrode. Figure 8 shows the conductivity of this modified electrode for various propylene concentration from 0.01 to 0.5 mol/L by CVs at the scan rate of 50 mV s ^−1^.

The propylene oxidation curves expose that anodic current peaks increase with expanding propylene concentration^50,51^. These obtained results confirm that our modified electrode SMWZCPE acts as an effective catalyst for the oxidation of propylene in 0.5 M H_2_SO_4_. Figure 8 inset demonstrates a linearity by:

$${\text{I}}_{{\text{p}}} \left( {\mu {\text{A}}} \right) = {23}.{27} + {\text{218 C}}\left( {{\text{r}}^{{2}} = 0.{9956}} \right).$$

It's suggested that the above relationship between I_p_ and [propylene] is owing to a diffusion-controlled mechanism.

#### Stability of SMWZCPE electrocatalyst

**Figure 9:**
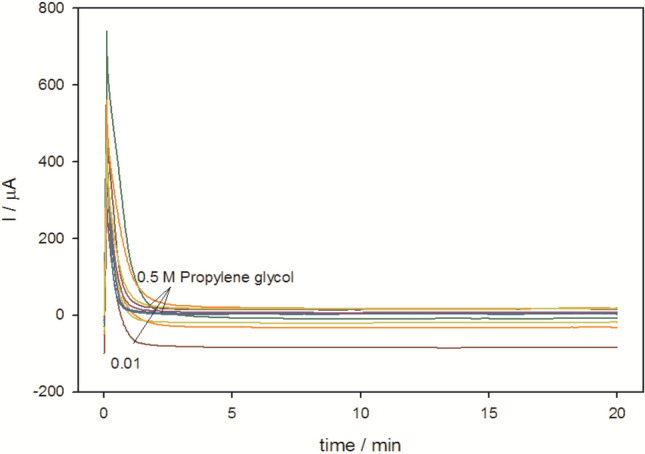
Chronoamperograms obtained at modified electrode SMWZCPE in 0.5 M H_2_SO_4_ using changed concentrations from (0.01–0.5 M) propylene glycol.

The stability of an electrocatalyst is an essential and evaluated by chronoamperomety. The chronoamperometric curves were obtained in the solution of 0.5 mol/L H_2_SO_4_ containing (0.01—0.5 mol/L ) propylene for 20 min at constant potential 0.7 V. Figure 9 demonstrates current—time relation for different concentrations of propylene glycol, in the first the modified electrode SMWZCPE reveals continous decay of anodic oxidation current and after ~ 2.5 min reached relatively stable value until the end of experiment (~ 20 min). This denotes good mechanical and electrocatalytic constancy of the modified electrode toward PG oxidation.

The comparison of current density for the electrochemical oxidation of different alcohols is given in Table 3 and it was found that our catalyst in this work gives the highest current density value in comparison to others as seen in the Table reaching to 0.135 mA cm^2^.

**Table 3:** The comparison of current density for the electrochemical oxidation of alcohol^a^.

Catalyst	Current in PG (mA cm^2^)	References
Pd/C in PG	0.013	^24^
Pd_68_Ni_32_/C in PG	0.108	^24^
Pd/C in EG	0.029	^24^
Pd_68_Ni_32_/C in EG	0.067	^24^
SMWZCPE in PG	0.135	This work

[a] PG is propylene glycol, EG is ethylene glycol.

Finally, based on the obtained outcomes its confirm that SMWZCPE modified electrode shows good promises to improve the activity of oxidation reaction of propylene glycol in acidic medium fuel cells.

## Conclusion

1. A novel environmental nano-catalyst successfully constructed by modified carbon paste electrode with zeolite, carbon nanotube and silver nanoparticles .
2. The characterization of the electrode SEM and EDX analysis shows that a well dispersed zeolite, CNT with the existence of silver nanoparticles occurs with a good percentage on the surface.
3. Electrochemical measurements discovered the exceptional electrocatalytic activity of nano-catalyst for oxidizing propylene.
4. Propylene oxidation progression was following diffusion – controlled process at electrocatalyst surface.
5. Enhancing CNT content developed the attained anodic peak current until greatest performance at the electrocatalyst comprising 40 mg of CNT.
6. The proposed SMWZCPE modified electrode shows acceptable stability towards propylene glycol oxidation.
